# Combined Hesperidin and Doxorubicin Treatment Induces Apoptosis and Modulates Inflammatory Cytokines in HeLa Cervical Cancer Cells

**DOI:** 10.3390/ijms26178753

**Published:** 2025-09-08

**Authors:** İlhan Özdemir, Yasemin Afşin, Mehmet Cudi Tuncer, Şamil Öztürk

**Affiliations:** 1 IVF Center, Private Buhara Hospital, Erzurum 25070, Turkey; ilhanozdemir25@yandex.com; 2 Gynecology and Obstetrics, Private Batman Life Hospital, Batman 72040, Turkey; dryaseminafsin@outlook.com; 3 Department of Anatomy, Faculty of Medicine, Dicle University, Diyarbakır 21200, Turkey; 4 Vocational School of Health Services, Çanakkale Onsekiz Mart University, Çanakkale 17100, Turkey; ozturksamil@outlook.com

**Keywords:** cervical cancer, hesperidin, doxorubicin, apoptosis, cytokines, HeLa cells, RT-qPCR, combination therapy

## Abstract

Cervical cancer is a major gynecological malignancy linked to hormonal dysregulation and genetic alterations. Chemotherapy is standard but limited by toxicity and chemoresistance, prompting interest in plant-derived adjuncts. This study examined the anticancer and immunomodulatory effects of Hesperidin (Hes), a citrus flavonoid, with Doxorubicin (DX) in HeLa cervical cancer cells. Cell viability was assessed by MTT assay, apoptotic markers (*Bcl-2*, *Caspase-3*) by RT-qPCR, and inflammatory cytokines (*IL-1β*, *IL-6*, *TNF-α*, *IFN-γ*) by ELISA. Cytokine levels were normalized to 10^4^ viable cells, and mRNA expression of all four cytokines was quantified by RT-qPCR, confirming protein-level changes and showing the strongest *IL-6* suppression with Hes+DX. Chou–Talalay combination index (CI) analysis demonstrated synergistic interactions (CI < 1.0) between Hes and DX across all tested concentrations, with strong synergism (CI < 0.7) at medium and high doses, particularly at 48 and 72 h. Hes alone showed dose-dependent cytotoxicity, while the combination markedly increased *Caspase-3*, reduced *Bcl-2*, and decreased *IL-1β*, *IL-6*, and *TNF-α*, indicating enhanced intrinsic apoptosis and complementary immunomodulation. These results suggest that Hes augments DX’s pro-apoptotic and anti-inflammatory effects, potentially allowing lower chemotherapy doses and reduced systemic toxicity in cervical cancer treatment.

## 1. Introduction

Cervical cancer is one of the most prevalent and lethal gynecological malignancies globally, with an estimated 600,000 new cases and over 340,000 deaths annually, particularly affecting women in developing countries. Persistent infection with high-risk human papillomavirus (HPV) types—particularly HPV-16 and HPV-18—is recognized as the principal etiological factor, although co-factors such as immunosuppression, long-term oral contraceptive use, smoking, and poor nutrition further contribute to disease progression [1]. While the implementation of HPV vaccination programs and improved screening techniques has significantly reduced the incidence of cervical cancer in high-income settings, the disease remains a substantial burden in regions with limited healthcare infrastructure [2].

HeLa cells, an immortal human cervical cancer cell line derived from the tumor of Henrietta Lacks in 1951, continue to serve as an essential model for cervical cancer research. Their capacity for continuous proliferation, coupled with a well-characterized genome, renders them highly suitable for evaluating the molecular mechanisms of cancer progression and drug response. HeLa cells are particularly valuable for studying apoptosis resistance, a hallmark of many advanced cancers, including cervical tumors [3].

Despite continuous advancements in oncology, chemotherapy remains a cornerstone of cervical cancer management, especially in advanced or recurrent cases. DX, an anthracycline antibiotic, is widely employed due to its potent antineoplastic effects. Its mechanism of action involves DNA intercalation, inhibition of topoisomerase II, and generation of reactive oxygen species, leading to cell cycle arrest and apoptosis [4]. However, the clinical use of DX is often limited by severe side effects, including cumulative dose-dependent cardiotoxicity, myelosuppression, and gastrointestinal toxicity [5]. Additionally, tumor cells frequently develop multidrug resistance via enhanced efflux pump activity, alterations in apoptotic gene expression, and metabolic adaptation, ultimately diminishing DX’s long-term efficacy [6].

To address these challenges, combination therapies that integrate natural compounds with conventional chemotherapeutics have garnered significant interest. Natural flavonoids, a large subclass of polyphenolic secondary metabolites found in fruits and vegetables, have demonstrated diverse biological activities, including antioxidant, anti-inflammatory, antiproliferative, antiangiogenic, and pro-apoptotic properties [7]. These bioactivities make flavonoids attractive candidates for cancer prevention and therapy, particularly due to their low toxicity profiles and ability to modulate intracellular signaling pathways involved in tumorigenesis [7,8].

Hes, a flavanone glycoside predominantly present in citrus fruits such as oranges and lemons, has emerged as a promising anticancer agent. Studies have shown that Hes can attenuate oxidative stress, inhibit cell proliferation, suppress angiogenesis, and activate mitochondrial-mediated apoptosis pathways in various cancer cell lines, including breast, lung, and colon cancers [8,9]. Its modulatory effects on apoptotic genes such as *Bcl-2* and *Caspase-3* suggest a potential to overcome apoptosis resistance, a critical feature of aggressive and drug-resistant cancers.

Given this background, the combination of Hes with established chemotherapeutic agents like DX may offer synergistic advantages: enhancing therapeutic efficacy, reducing required drug dosages, and minimizing systemic toxicity. However, the underlying molecular mechanisms of this potential synergy in cervical cancer models remain insufficiently characterized.

Therefore, the present study was designed to evaluate the combined cytotoxic, pro-apoptotic, and immunomodulatory effects of Hes and DX in HeLa cervical cancer cells. Specifically, we assessed cell viability, quantified apoptotic regulators (*Bcl-2* and *Caspase-3*) at the mRNA level, and analyzed inflammatory cytokine profiles (*IL-1β*, *IL-6*, *TNF-α*, *IFN-γ*) at both protein and transcriptional levels, with cytokine concentrations normalized to viable cell counts. The synergistic interaction between Hes and DX was further quantified using the Chou–Talalay CI method. Our findings provide mechanistic and quantitative evidence supporting this combination as a potential adjuvant strategy for enhancing apoptosis while attenuating pro-inflammatory responses in cervical cancer therapy.

## 2. Results

### 2.1. Cell Viability and Cytotoxicity

**Figure 1:**
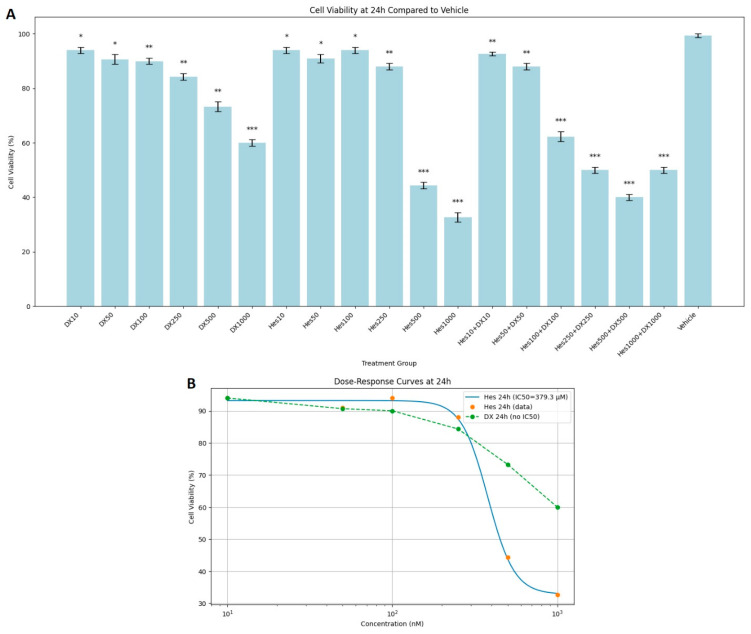
(**A**) Cytotoxic effects of Hes and DX on HeLa cells after 24 h of treatment. Cell viability is expressed as a percentage relative to the vehicle control group (Vehicle = 100%). HeLa cells were treated with increasing concentrations of Hes (10–1000 µM) and DX (10–1000 nM), and viability was measured using the MTT assay. (**B**) At 24 h, cytotoxic effects of DX were minimal, and an IC_50_ value could not be determined. However, Hes treatment resulted in a measurable IC_50_ value of 379.3 µM. Data are presented as mean ± standard deviation (SD), *n* = 3. * *p* < 0.05, ** *p* < 0.01, *** *p* < 0.001, compared with the vehicle control.

MTT test results showed that Hes, DX, and their combinations applied as treatments for 24, 48, and 72 h caused a significant decrease in the viability of HeLa cervical cancer cells in a dose- and time-dependent manner. At the end of 24 h incubation, cell viability was observed as 100% in the control group. While the viability was between 92 and 96% in low dose Hes (Hes10–Hes100) groups, a significant decrease was observed at high doses (Hes500: 44%, Hes1000: 33%). Similarly, high doses of DX (DX500: 73%, DX1000: 60%) also caused a significant decrease in viability. A more significant decrease was observed in Hes and DX combinations; in particular, cell viability decreased to 40% in the Hes500+DX500 group and to 50% in the Hes1000+DX1000 group (Figure 1A). A statistically significant decrease in viability was observed at 25 nM DX compared with the vehicle (Figure 1A). DX treatment did not yield a measurable IC_50_ value at the 24 h time point. For Hes treatment, IC_50_ values were determined as 379.3 µM at 24 h (Figure 1B).

**Figure 2:**
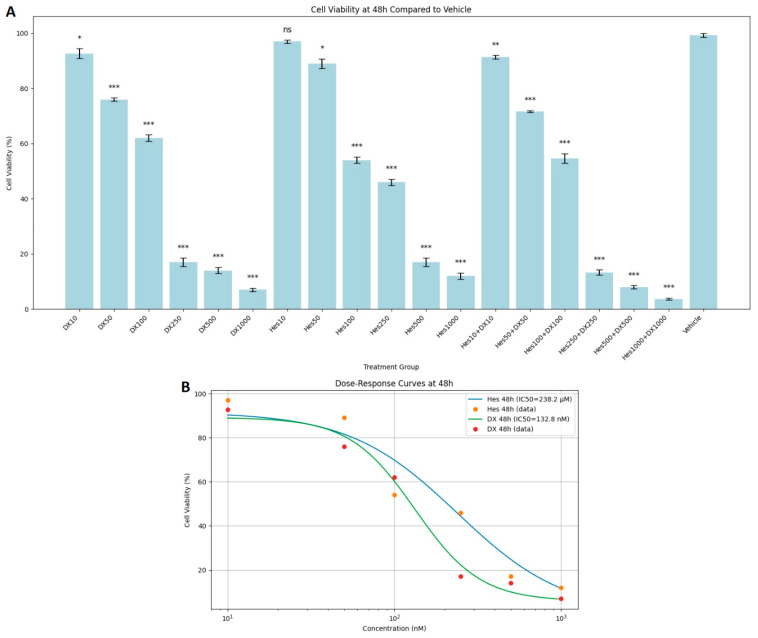
(**A**) Cytotoxic effects of Hes and DX on HeLa cells after 48 h of treatment. Cell viability is expressed as a percentage relative to the vehicle control group (Vehicle = 100%). HeLa cells were treated with Hes (10–1000 µM) and DX (10–1000 nM), and viability was assessed using the MTT assay. (**B**) A dose- and time-dependent decrease in cell viability was observed, with IC_50_ values calculated as 132.8 nM for DX and 238.2 µM for Hes at 48 h. Data are presented as mean ± standard deviation (SD), *n* = 3. * *p* < 0.05, ** *p* < 0.01, *** *p* < 0.001, ns: not significant, compared with the vehicle control.

At 48 h incubation, Hes and DX applications alone reduced cell viability even more. Significant toxicity was observed especially at Hes500 (16%), Hes1000 (12%), DX500 (14%), and DX1000 (7%) doses. A synergistic effect was noted in the combination groups. Cell viability was determined as 13%, 8%, and 4% in Hes250+DX250, Hes500+DX500, and Hes1000+DX1000 groups, respectively (Figure 2A). In addition, DX and Hes IC_50_ values were determined as 132.8 nM and 238.4 µM at 48 h (Figure 2B).

**Figure 3:**
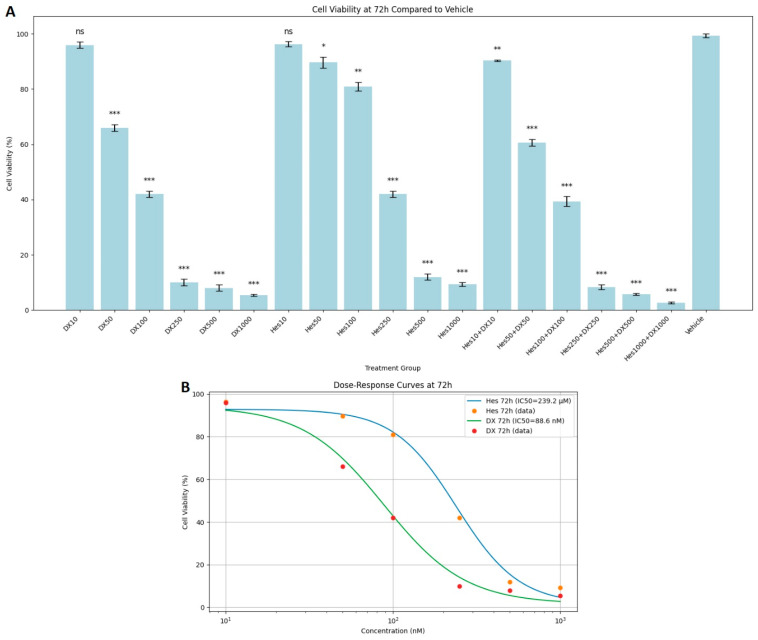
(**A**) Cytotoxic effects of Hes and DX on HeLa cells after 72 h of treatment. Cell viability is expressed as a percentage relative to the vehicle control group (Vehicle = 100%). HeLa cells were treated with Hes (10–1000 µM) and DX (10–1000 nM), and viability was assessed using the MTT assay. (**B**) A dose- and time-dependent decrease in cell viability was observed, with IC_50_ values calculated as 88.6 nM for DX and 239.2 µM for Hes at 72 h. Data are presented as mean ± standard deviation (SD), *n* = 3. * *p* < 0.05, ** *p* < 0.01, *** *p* < 0.001, ns: not significant, compared with the vehicle control.

In the 72 h results, the decrease in viability rates became most obvious. Especially in the high-dose combination groups (Hes500+DX500: 5%, Hes1000+DX1000: 3%), almost complete suppression of cell viability was observed. The combined application of Hes and DX produced a stronger antiproliferative effect compared with their single applications (Figure 3A). In experiments cultured with 100,000 cells, the average cell number in the 1000 nM DX group was approximately 65,000 across all time points, and this further declined to 49.7 after 72 h of exposure (Figure 3A). DX and Hes IC_50_ values were determined as 88.6 nM and 239.2 µM at 72 h (Figure 3B). Viability significantly declined at concentrations near or exceeding these IC_50_ values. When comparing the cytotoxic effects of Hes and DX, the DX-treated groups exhibited a more pronounced reduction in HeLa cell viability at equivalent time points.

### 2.2. Chou–Talalay CI Findings

The CI values calculated for the combination of Hes and DX at 24, 48, and 72 h are presented in Table 1. At all tested time points, the combination treatments demonstrated CI values below 1.0, indicating synergistic interactions. Strong synergism (CI < 0.7) was observed at medium and high dose combinations, particularly at 48 and 72 h, which corresponded to the most pronounced reduction in cell viability. Low dose combinations exhibited moderate synergism (0.7 ≤ CI < 0.9) at earlier time points. No antagonistic effects (CI > 1.0) were detected in any of the tested combinations. These results corroborate the MTT assay findings, supporting that the combined treatment of Hes and DX exerts a synergistic inhibitory effect on HeLa cervical cancer cell viability in a dose- and time-dependent manner.

### 2.3. Inflammatory Cytokine Levels (Normalized to Viable Cell Count)

**Figure 4:**
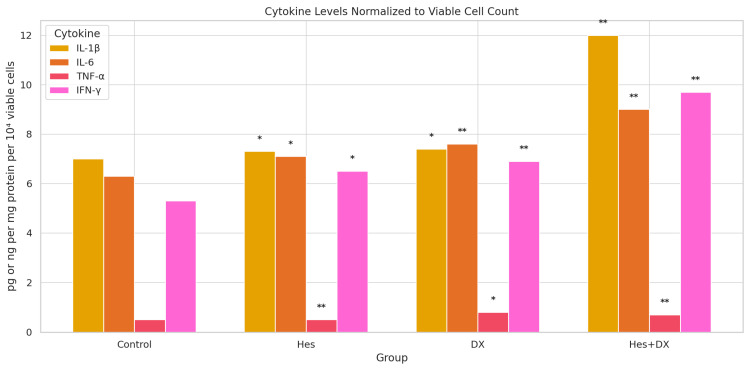
Normalization of proinflammatory cytokine levels to viable cell count in HeLa cells after treatment with Hes and DX. Bar graphs represent the levels of *IL-1β*, *IL-6*, *TNF-α*, and *IFN-γ* in culture supernatants of HeLa cells treated with Hes, DX, and Hes + DX, compared with untreated control cells. Cytokine levels were normalized to viable cell count and are expressed as pg per 10^4^ viable cells. Data are presented as mean ± standard deviation (SD), *n* = 3. Statistical analysis was performed using one-way ANOVA followed by Tukey’s post hoc test. Significant differences between groups are indicated as follows: * *p* < 0.05, ** *p* < 0.01.

To account for differences in cell proliferation among treatment groups, cytokine secretion levels were normalized to viable cell counts and expressed as pg per 10^4^ viable cells (Figure 4). One-way ANOVA revealed statistically significant differences among the treatment groups for all cytokines (*p* < 0.05), which were further examined by Tukey’s post hoc analysis.

For *IL-1β*, Hes and DX monotherapies resulted in modest increases compared with control, while the Hes+DX combination significantly elevated *IL-1β* levels relative to control and both single treatments (*p* < 0.01).

For *IL-6*, a similar trend was observed, with Hes and DX producing higher normalized secretion compared with control, and the Hes+DX group showing the highest *IL-6* level among all groups (*p* < 0.01).

In contrast, *TNF-α* secretion was markedly suppressed by Hes (*p* < 0.01) and further reduced in the Hes+DX group (*p* < 0.01 vs. control), whereas DX monotherapy significantly lowered *TNF-α* relative to control (*p* < 0.05).

For *IFN-γ*, both Hes and DX treatments significantly increased secretion relative to control, with the highest levels detected in the Hes+DX group (*p* < 0.01).

These findings, after normalization to viable cell counts, suggest that the combination of Hes and DX modulates cytokine secretion in a pattern distinct from protein-level normalization, potentially reflecting treatment-induced changes in both cell viability and inflammatory signaling.

### 2.4. qRT-PCR Validation of Cytokine Expression

**Figure 5:**
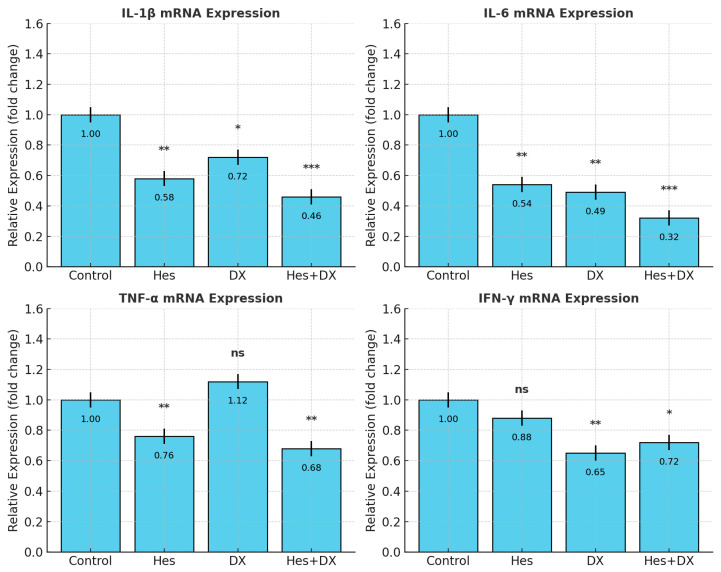
Relative mRNA expression levels of *IL-1β*, *IL-6*, *TNF-α*, and *IFN-γ* in HeLa cells after treatment with Hes, DX, or their combination (Hes+DX), as determined by RT-qPCR. Cytokine expression was normalized to GAPDH and presented as fold change relative to the control group. Data are shown as mean ± SD (*n* = 3). *p* < 0.05 (*), *p* < 0.01 (**), and *p* < 0.001 (***) indicate statistically significant differences compared with the control group, as determined by one-way ANOVA followed by Tukey’s post hoc test. ns: non-significant.

To confirm whether changes in cytokine secretion reflected transcriptional regulation, mRNA levels of *IL-1β*, *IL-6*, *TNF-α*, and *IFN-γ* were quantified by RT-qPCR (Figure 5). Consistent with ELISA data normalized to viable cell counts, Hes and DX significantly reduced *IL-6* transcript abundance compared with control (*p* < 0.001), with the most pronounced reduction in the Hes+DX group (*p* < 0.001). *IL-1β* mRNA levels were decreased by Hes (*p* < 0.01) and further reduced in the Hes+DX group (*p* < 0.001), while DX caused a modest decrease (*p* < 0.05). *TNF-α* transcripts were lowered by Hes and Hes+DX (*p* < 0.01), but were unchanged in the DX group. *IFN-γ* mRNA was reduced in the DX (*p* < 0.01) and Hes+DX (*p* < 0.05) groups, with no significant change in the Hes monotherapy group. These findings confirm that the observed cytokine modulation involves transcriptional regulation and supports the protein-level results.

### 2.5. Caspase-3/7 Levels

**Figure 6:**
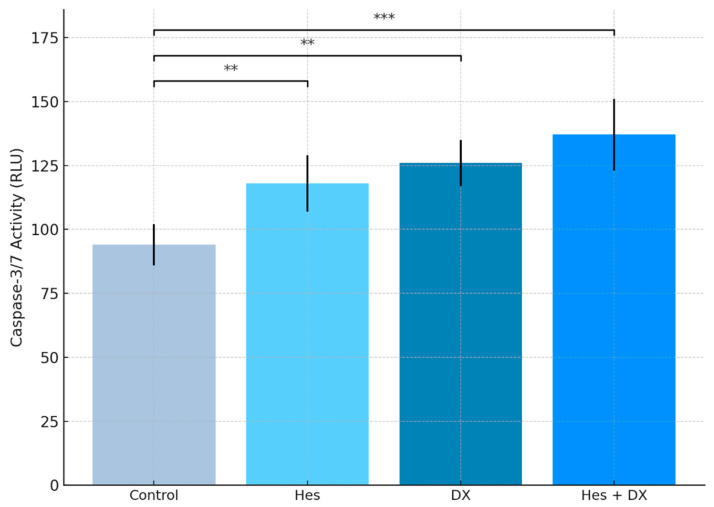
*Caspase-3/7* activity in HeLa cells following 48 h treatment with IC_50_ concentrations of Hes, DX, and their combination. Caspase activity was quantified using a luminescence-based Caspase-Glo^®^ 3/7 assay. A significant increase in *caspase-3/7* activity was observed in all treatment groups compared with the control, with the highest activity detected in the Hes + DX combination group. Treatments were applied at IC_50_ concentrations (Hes: 238.2 µM; DX: 132.8 nM). Data are presented as mean ± standard deviation (SD), *n* = 3. Statistical significance was determined using one-way ANOVA followed by Tukey’s post hoc test: ** *p* < 0.01, *** *p* < 0.001 versus control.

*Caspase-3/7* activity was measured to evaluate the apoptosis induction in HeLa cells by Hes and DX. The data obtained showed that when both agents were applied alone, there was a significant increase in caspase-3/7 levels (*p* < 0.01). While an approximately 25% increase in *caspase-3/7* activity was observed in the Hes group compared with the control group, this increase was around 34% in the DX group. The highest *caspase-3/7* activity was detected in the combined treatment group (Hes + DX), and this increase was statistically highly significant compared with the control group (*p* < 0.001). These results indicate that Hes has a synergistic effect in increasing apoptosis when applied together with DX (Figure 6).

### 2.6. qRT-PCR Findings

Quantitative real-time PCR (qRT-PCR) was performed on nine independent samples representing the control, Hes, DX, and Hes + DX treatment groups. The expression levels of the apoptotic markers *Bcl-2* and *Caspase-3* were normalized to β-actin, which served as the internal reference gene.

**Figure 7:**
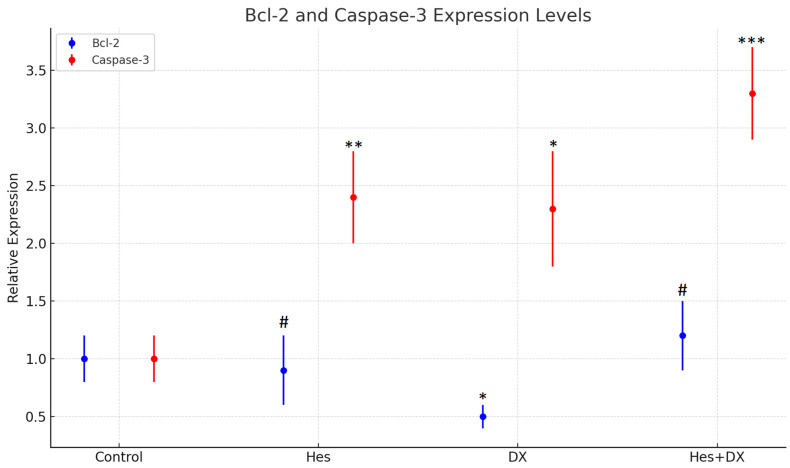
*Bcl-2* and *Caspase-3* gene expression levels in HeLa cells after 48 h treatment with Hes, DX, and their combination (*n* = 3, mean ± SD). Statistical analysis was performed using one-way ANOVA followed by Tukey’s HSD post hoc test. Significant comparisons: Hes vs. Control for *Caspase-3* (** *p* = 0.01); DX vs. Control for *Bcl-2* (* *p* = 0.03); DX vs. Control for *Caspase-3* (* *p* = 0.02); Hes + DX vs. Control for *Caspase-3* (*** *p* = 0.001). There was no statistical difference between Control and Hes, Control and Hes+DX (# *p* > 0.05).

At the 48 h time point, *Bcl-2* expression was found to be decreased in both the DX and Hes groups compared with control; however, the reduction did not reach statistical significance in the DX group. A significant decrease in *Bcl-2* expression was observed when comparing the Hes group to the Hes + DX combination group (*p* < 0.05), indicating enhanced suppression of anti-apoptotic signaling when the two agents were administered together (Figure 7).

*Caspase-3* expression, in contrast, was upregulated in all treatment groups relative to control. The highest expression level was observed in the Hes + DX group, where *Caspase-3* showed a five-fold increase. This upregulation was statistically significant in all treatment groups compared with the control (*p* < 0.01). However, there was no significant difference in *Caspase-3* expression between the DX and Hes monotherapy groups or between the monotherapies and the combination group, suggesting a plateau effect in gene activation (Figure 7).

These findings indicate that while both Hes and DX modulate apoptotic gene expression individually, their combination exerts a more potent effect, particularly on *Caspase-3* activation. The elevated expression of *Caspase-3* and concurrent suppression of *Bcl-2* are consistent with activation of the intrinsic apoptotic pathway. Given that HeLa cells are known for their active anti-apoptotic mechanisms and resistance to cell death, the observed gene expression shifts suggest that the Hes + DX combination may effectively overcome apoptosis resistance in this cervical cancer model.

### 2.7. Enrichment Apoptotic Pathway Analysis

**Figure 8:**
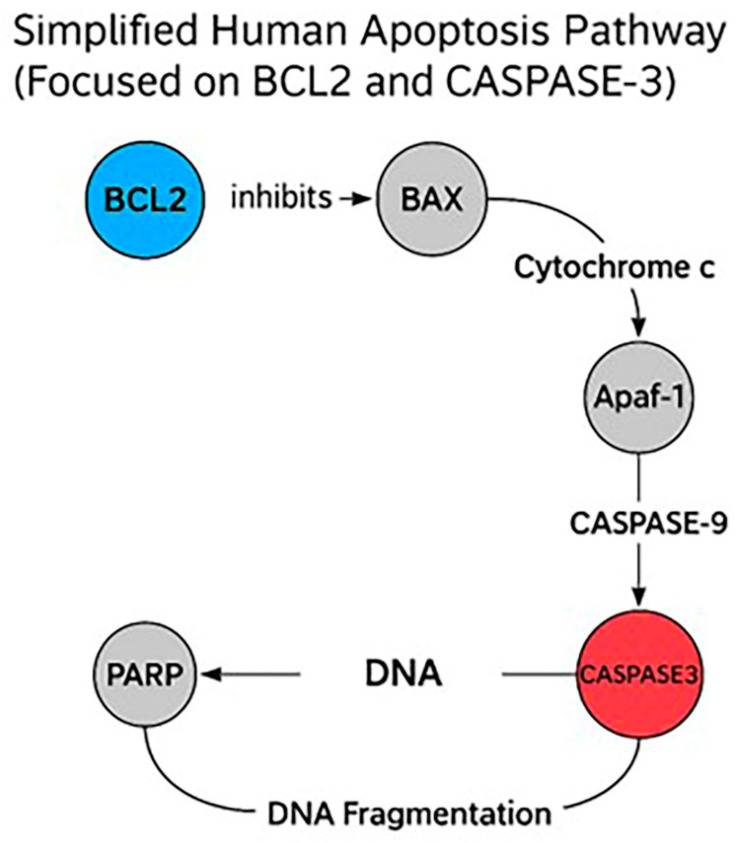
The diagram illustrates the mitochondrial (intrinsic) apoptotic signaling cascade, emphasizing the pharmacological targets *Bcl-2* and *Caspase-3*. In the absence of apoptotic stimuli, *Bcl-2* (blue) stabilizes the mitochondrial membrane by inhibiting BAX activation and preventing cytochrome c release. Upon pro-apoptotic signaling or drug-induced stress, Bax oligomerizes to promote MOMP, leading to cytochrome c release into the cytosol. Cytochrome c interacts with Apaf-1 to form the apoptosome, which activates caspase-9, subsequently triggering the cleavage of executioner *Caspase-3* (red). Activated *Caspase-3* cleaves substrates such as PARP, culminating in DNA fragmentation and cell death. Agents like Hes and DX enhance apoptosis by downregulating *Bcl-2* and upregulating *Caspase-3* activity, thereby restoring the mitochondrial apoptotic machinery in apoptosis-resistant cancer cells such as HeLa. Apaf-1: Apoptotic Protease Activating Factor 1; PARP: Poly (ADP-ribose) Polymerase.

Reactome pathway enrichment analysis revealed significant modulation of the intrinsic apoptotic signaling cascade in HeLa cells treated with Hes and DX. The expression profiles of *Caspase-3* and *Bcl-2*, two central regulators of mitochondrial apoptosis, were directly implicated in this enrichment. *Caspase-3* was significantly upregulated, while *Bcl-2* was markedly downregulated following combination treatment, consistent with the activation of apoptosis at the molecular level (Figure 8).

These genes were enriched in critical pathways such as the “Intrinsic Pathway for Apoptosis” and the “Execution Phase of Apoptosis,” both of which are central to mitochondrial-mediated cell death. In this context, reduced *Bcl-2* expression likely facilitated the destabilization of the mitochondrial membrane and the release of cytochrome c, promoting apoptosome formation and subsequent activation of *Caspase-3*.

These findings suggest that the combination of Hes and DX reactivates intrinsic apoptotic signaling in HeLa cells by simultaneously suppressing anti-apoptotic defenses and enhancing caspase-mediated cell death, thereby contributing to the observed increase in cytotoxicity.

### 2.8. Reactome Pathway Analysis

Pathway enrichment analysis was conducted to investigate the roles of *Bcl-2* and *Caspase-3* in Hes- and DX-treated HeLa cells. *Bcl-2* is a critical anti-apoptotic protein that prevents mitochondrial outer membrane permeabilization, thereby inhibiting cytochrome c release and downstream caspase activation. In contrast, *Caspase-3* is a key executioner protease activated during the late phase of intrinsic apoptosis, responsible for the cleavage of various cellular substrates and the execution of cell death.

According to Reactome pathway analysis, both *Bcl-2* and *Caspase-3* are involved in essential mitochondrial-mediated apoptotic pathways, including “Regulation of Apoptosis,” “Intrinsic Pathway for Apoptosis,” “Caspase Activation,” and “Execution Phase of Apoptosis.” Treatment with Hes alone led to the downregulation of *Bcl-2* and upregulation of *Caspase-3*, indicating partial activation of the apoptotic cascade. However, the combination of Hes and DX enhanced this effect synergistically, resulting in a more pronounced decrease in *Bcl-2* and increase in *Caspase-3* expression, suggesting a robust activation of mitochondrial apoptosis.

**Figure 9:**
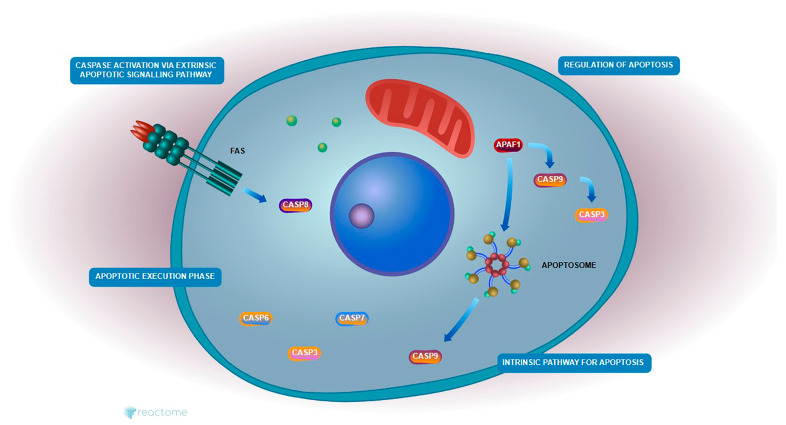
Schematic representation of the extrinsic and intrinsic apoptotic pathways based on Reactome analysis. The extrinsic pathway is initiated by activation of death receptors such as Fas, leading to recruitment and activation of *Caspase-8*, which subsequently activates downstream executioner caspases including *Caspase-3*. The intrinsic (mitochondrial) pathway is triggered by intracellular stress signals, resulting in the release of cytochrome c from mitochondria, formation of the apoptosome complex via Apaf-1 and *Caspase-9*, and activation of *Caspase-3*. Both pathways converge on *Caspase-3*, leading to the execution of apoptosis. In this study, *Caspase-3* and *Caspase-9* were identified as key molecular targets modulated by Hes and DX treatment.

These findings support the hypothesis that the combination therapy facilitates intrinsic apoptotic signaling by simultaneously repressing anti-apoptotic regulators and enhancing pro-apoptotic execution mechanisms. This mechanistic insight is visually summarized in Figure 9.

### 2.9. Gene Ontology

To further explore the functional roles of *Bcl-2* and *Caspase-3*, Gene Ontology (GO) enrichment analysis was conducted within the Biological Process (BP) category. The analysis revealed a strong association of these genes with key apoptosis-related processes.

The top enriched GO terms included

- Intrinsic apoptotic signaling pathway in response to hypoxia
- Regulation of mitochondrial membrane permeability involved in apoptotic process
- Execution phase of apoptosis
- B cell negative selection
- Dendritic cell apoptotic process

These findings underscore the functional diversity of *Bcl-2* and *Caspase-3* in regulating cell death pathways, particularly in the context of mitochondrial dysfunction and immune-mediated apoptosis. Notably, their involvement in hypoxia-induced and immune cell apoptosis further supports their relevance in the tumor microenvironment of cervical cancer.

**Figure 10:**
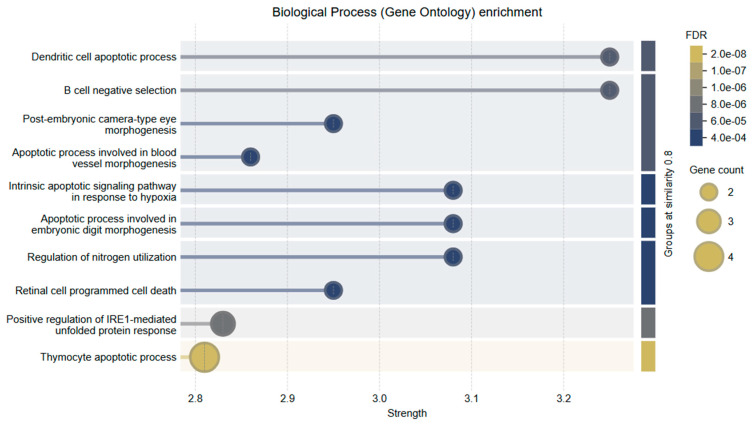
GO Biological Process (BP) enrichment analysis of apoptosis-related genes. The bubble plot displays the top enriched biological processes ranked by strength and false discovery rate (FDR). Key enriched processes include dendritic cell apoptotic process, B cell negative selection, intrinsic apoptotic signaling in response to hypoxia, and thymocyte apoptotic process. Bubble size indicates the number of genes associated with each pathway, while bubble color represents FDR values, with darker shades indicating stronger statistical significance.

The combined treatment of Hes and DX modulated these biological processes more effectively than either agent alone, reinforcing the synergistic activation of intrinsic apoptotic signaling. A visual summary of these enriched biological processes and their relationship to mitochondrial apoptosis is presented in Figure 10.

Tissue-specific expression analysis revealed that the genes evaluated in this study, particularly *Bcl-2* and *Caspase-3*, are significantly enriched in tissues of the female reproductive system, including the uterus, ovaries, and cervical epithelium. These results support the biological validity of using HeLa cells as a cervical cancer model and further substantiate the functional relevance of the selected genes to the disease context. *Bcl-2* expression was markedly elevated in endometrial and cervical epithelial tissues. This is consistent with its anti-apoptotic role and suggests that *Bcl-2* may contribute to enhanced cell survival and tumor progression in these tissues by inhibiting programmed cell death.

*Caspase-3* demonstrated moderate to high expression across various tissue types, with notable abundance in the cervix and uterine tissues. As a key executioner caspase in the intrinsic apoptotic pathway, its expression in these regions suggests that it may play an essential role in regulating local tumor cell apoptosis, especially in response to therapeutic agents.

**Figure 11:**
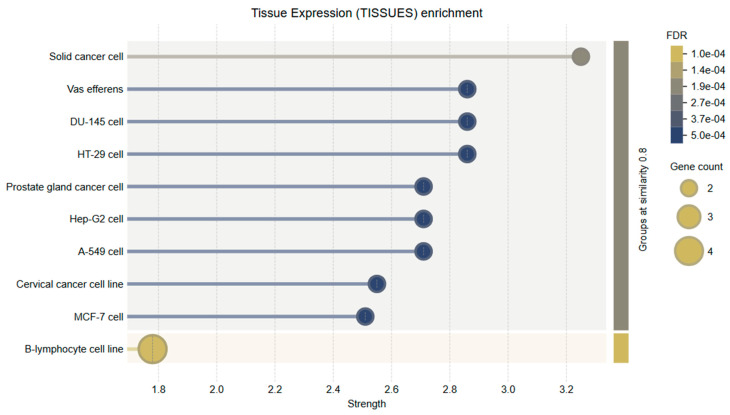
Tissue expression enrichment analysis of target genes. This bubble plot visualizes the tissue-specific expression of apoptosis-related genes, particularly *Bcl-2* and *Caspase-3*, across various cell lines. Notable enrichment was observed in cervical cancer cell lines, aligning with the HeLa model used in this study. Other enriched tissues include MCF-7 (breast cancer), A-549 (lung cancer), and prostate gland cancer cells, reflecting the broader relevance of these genes in solid tumors. Bubble size indicates the number of genes expressed, while the color gradient represents the FDR, with darker shades indicating stronger statistical significance.

These findings highlight the tissue-specific biological significance of *Bcl-2* and *Caspase-3* in cervical tissue and underscore the potential of natural compounds such as Hes to modulate apoptosis-related mechanisms in gynecologic cancers (Figure 11).

## 3. Discussion

Cancer remains one of the leading causes of morbidity and mortality worldwide, posing a major challenge to modern medicine and public health systems. Its increasing incidence is strongly associated with environmental factors such as exposure to radiation, unhealthy lifestyle habits, and genetic susceptibility. Chemotherapy, one of the primary treatment modalities, aims to halt the uncontrolled proliferation of cancer cells by disrupting cell division and inducing cytotoxicity. However, its use is frequently limited by adverse effects, multidrug resistance, and lack of selectivity between healthy and malignant cells. DX, an anthracycline antibiotic, is a widely used chemotherapeutic agent that functions by intercalating into DNA and stabilizing the DNA-topoisomerase II complex, thereby inhibiting replication and promoting apoptosis. Despite its effectiveness, DX is associated with dose-dependent cardiotoxicity and resistance mechanisms in tumor cells [10]. Consequently, there is growing interest in adjuvant or alternative therapies based on natural compounds with high therapeutic indices and fewer side effects. Among these, flavonoids such as Hes, found abundantly in citrus fruits, have demonstrated antioxidant, anti-inflammatory, and antiproliferative properties in a variety of tumor models [11,12].

In the present study, we evaluated the combined apoptotic effects of Hes and DX on HeLa cervical cancer cells. Cell proliferation was assessed via MTT assays, and apoptotic gene expression was quantified using RT-qPCR. Hes alone showed cytotoxic and pro-apoptotic effects, which were significantly enhanced when co-administered with DX. This synergistic interaction resulted in upregulation of *Caspase-3* and downregulation of *Bcl-2* expression, confirming enhanced apoptotic activity. These findings support the potential of Hes as a complementary agent in cervical cancer therapy, particularly when used in combination with conventional chemotherapeutics like DX [13]. Importantly, although DX induces apoptosis partly through the generation of reactive oxygen species (ROS), co-treatment with Hes, a compound known for its antioxidant properties, did not hinder its cytotoxic effects. Instead, Hes appeared to enhance the therapeutic efficacy while potentially mitigating DX-induced inflammation and oxidative damage, which is particularly relevant for protecting non-malignant cells. This suggests a context-dependent antioxidant/pro-oxidant duality in cancer therapy. Although the use of a ROS-producing chemotherapeutic such as DX and antioxidant Hes together seems paradoxical, this study revealed that Hes may synergistically enhance the apoptosis-inducing effect rather than inhibiting DX-induced ROS production. In addition, the antioxidant capacity of Hes has the potential to improve the side effect profile by reducing the oxidative damage caused by DX in normal cells.

Flavones, a subclass of flavonoids and key constituents of the secondary metabolite group, represent some of the most important bioactive compounds in nature [14]. They possess a wide range of pharmacological activities, including anti-inflammatory, antioxidant, anticarcinogenic, antiproliferative, and antiangiogenic effects, with minimal toxicity [15]. Owing to this therapeutic versatility, flavones have emerged as valuable targets in drug discovery and development. Their potential role in cancer prevention and treatment is particularly notable, given their ability to selectively act on malignant cells with limited impact on normal tissue [16]. Flavonoids, broadly distributed in nature, have demonstrated high therapeutic efficacy and are considered promising candidates for cancer therapy. Their mechanisms of action involve inhibition of proliferation and metastasis, along with the promotion of apoptosis through the modulation of various intracellular signaling cascades [17,18]. Apoptosis, a form of programmed cell death, is tightly regulated by several gene families, including those encoding *Bcl-2* and *Caspase-3*. In the present study, we investigated the expression of these key apoptotic genes in HeLa cervical cancer cells following treatment with Hes, DX, and their combination. Our results demonstrated that *Bcl-2* expression was decreased in both the Hes and DX groups compared with control, and was further suppressed in the Hes+DX combination group, indicating a synergistic inhibition of anti-apoptotic signaling. In contrast, *Caspase-3* expression was significantly upregulated in all experimental groups, with the highest increase observed in the combination group.

The caspase cascade plays a central role in the execution of apoptosis. Our findings showed that Hes significantly enhanced *Caspase-3* expression in HeLa cells, indicating activation of the intrinsic apoptotic pathway. Importantly, the *Bcl-2/Caspase-3* expression ratio is known to be a critical determinant in the regulation of apoptosis [19]. The observed alterations in this ratio suggest a shift toward apoptotic activation in cells treated with either Hes or DX. Moreover, p53, a tumor suppressor protein, regulates *Bcl-2* expression, mediates DNA repair, and governs cell cycle arrest and apoptosis. DNA-damaging agents, such as DX, are known to activate p53, which in turn induces the expression of pro-apoptotic genes like Bax and downregulates anti-apoptotic members such as *Bcl-2* [20,21]. In our study, Hes also contributed to increased *Caspase-3* expression, further supporting its pro-apoptotic role.

Reactome pathway enrichment analysis revealed significant involvement of the evaluated genes in several critical apoptotic processes, including the “Intrinsic Apoptotic Signaling Pathway,” “Caspase Activation,” and the “Execution Phase of Apoptosis.” Gene expression data supported this, showing decreased *Bcl*-2 and increased *Caspase-3* levels, particularly after Hes treatment. These findings confirm that Hes induces mitochondrial-mediated apoptosis. Notably, in the combination group (Hes+DX), *Bcl-2* suppression and *Caspase*-3 activation were more pronounced, suggesting a synergistic apoptotic response. Visualization of this pathway demonstrated that Hes weakens *Bcl-2*-mediated inhibition of MOMP, thereby promoting cytochrome c release and subsequent caspase activation. Collectively, these results indicate that Hes possesses significant therapeutic potential by enhancing intrinsic apoptotic mechanisms in cervical cancer cells [22,23]. The Reactome and GO analyses used in this study should be considered as hypothesis-generating approaches that require experimental validation. In future studies, translational examination of signaling proteins (e.g., Apaf-1, Caspase-9, Bax) involved in these pathways will enable stronger mechanistic connections to be established. Our study additionally confirmed the modulation of cytokine secretion at the transcriptional level through RT-qPCR analysis, which revealed significant downregulation of *IL-1β*, *IL-6*, *TNF-α*, and *IFN-γ* mRNA expression, particularly in the Hes+DX group. These transcriptional changes were consistent with ELISA findings normalized to viable cell counts, thereby reinforcing the robustness of our results and reducing the likelihood that observed protein-level differences were due to post-transcriptional regulation alone. The suppression of pro-inflammatory cytokines at both mRNA and protein levels suggests that the Hes+DX combination not only enhances apoptotic signaling but also exerts a complementary anti-inflammatory effect. This dual modulation of apoptosis and inflammation could be particularly advantageous in a clinical context, as it may promote tumor cell elimination while mitigating treatment-associated inflammatory responses.

In our study, the combination of Hes and DX elicited the highest level of *Caspase-3* expression, reinforcing the synergistic nature of their pro-apoptotic effect. Meanwhile, *Bcl-2* expression was markedly reduced in both monotherapy groups. This synergism is consistent with previous studies reporting similar results in human ovarian carcinoma cells [24]. Moreover, parallel pro-apoptotic effects have been demonstrated with curcumin treatment in cervical cancer models [25,26,27]. Hes has also shown potent apoptotic effects in osteosarcoma models. In a study involving drug-sensitive and resistant cancer cell lines, Gallic acid was reported to enhance G2/M phase arrest when combined with paclitaxel, potentially due to the pro-oxidative stress induced by flavonoids and the complementary extracellular signaling effects of paclitaxel [24,25]. In our study, MTT analysis confirmed that Hes and DX co-treatment significantly increased cytotoxicity, while RT-qPCR analysis revealed enhanced apoptosis through the modulation of *Bcl-2* and *Caspase-3* gene expression. The Chou–Talalay CI analysis further confirmed the synergistic interaction between Hes and DX in HeLa cervical cancer cells. CI values were consistently below 1.0 at all tested time points, indicating synergism, with the strongest effects (CI < 0.7) observed at medium and high dose combinations, particularly at 48 and 72 h. These findings align with the marked reduction in cell viability and the significant modulation of apoptotic and inflammatory markers observed in this study. The observed synergism is likely attributable to complementary mechanisms, where Hes enhances DX-induced intrinsic apoptosis by upregulating *Caspase-3* and downregulating *Bcl-2*, while concurrently attenuating pro-inflammatory cytokines such as *IL-1β*, *IL-6*, and *TNF-α*. This dual action may contribute to a more favorable tumor microenvironment and sensitize cancer cells to chemotherapeutic-induced apoptosis. Comparable synergistic effects have been reported for other flavonoid–chemotherapy combinations, suggesting that polyphenol-mediated modulation of apoptotic and inflammatory pathways is a key factor in enhancing chemotherapy efficacy. Clinically, such synergy could permit the use of lower DX doses, potentially reducing systemic toxicity while maintaining or improving therapeutic outcomes.

Recent studies have highlighted multiple intracellular mechanisms by which Hes exerts its anticancer effects in HeLa cervical cancer cells, further supporting the findings of the present study. Pandey et al. [28] demonstrated that Hes treatment induces apoptosis in HeLa cells by targeting the oncoprotein Jab1, leading to its downregulation and concurrent upregulation of p27, a key cell cycle inhibitor. This apoptotic induction was closely associated with increased reactive oxygen species generation and *Caspase-3* activation. Consistently, our study showed that Hes monotherapy significantly elevated *Caspase-3* gene expression and *Caspase-3/7* enzymatic activity, suggesting that ROS-mediated mitochondrial pathway activation may underlie this pro-apoptotic response. Moreover, our combination treatment group exhibited the highest *Caspase-3* expression and activity, implying that modulation of the Jab1–p27 axis may act synergistically with other apoptotic signals enhanced by DX to overcome resistance mechanisms in HeLa cells.

Similarly, Wang et al. [29] provided evidence that Hes induces apoptosis in HeLa cells through endoplasmic reticulum stress-mediated mechanisms, as shown by increased expression of GADD153 and GRP78 proteins, which are hallmark indicators of ER stress. Their study also reported mitochondrial dysfunction characterized by increased intracellular calcium levels, cytochrome c release, and loss of mitochondrial membrane potential, ultimately resulting in *Caspase-3* activation. Although ER stress markers were not assessed in our study, the significant downregulation of *Bcl-2* and robust *Caspase-3* upregulation we observed strongly aligned with the activation of the intrinsic mitochondrial apoptotic pathway, which may operate downstream of ER stress. These parallels suggest that Hes may simultaneously trigger both ER-dependent and mitochondrial-mediated apoptosis, converging on *Caspase-3* activation, a central effector confirmed in both our study and the previous literature.

Taken together, our findings are consistent with prior research and extend the mechanistic understanding by demonstrating that Hes not only induces apoptosis via mitochondrial mechanisms but also potentiates DX-induced cytotoxicity. This dual action may provide a therapeutic advantage by lowering the required chemotherapeutic dose and reducing associated systemic toxicity. Further investigation is warranted to validate whether Jab1 inhibition and ER stress induction occur in parallel with *Bcl-2* and *Caspase-3* modulation in combined Hes and DX treatment regimens.

Consistent with our findings, Korga et al. [30] demonstrated that hesperidin significantly augmented the cytotoxic effects of doxorubicin in HepG2 liver cancer cells by modulating glycolytic gene expression and enhancing double-strand DNA breaks and ROS-related damage, suggesting a strong synergistic interaction not solely dependent on oxidative stress mechanisms. Importantly, their results support the idea that hesperidin can sensitize tumor cells to doxorubicin via alternative intracellular mechanisms that could be exploited in chemotherapeutic strategies. Furthermore, an earlier study by Kusharyanti et al. [31] reported that the combination of hesperidin and doxorubicin in HeLa cervical cancer cells resulted in significant downregulation of *Bcl-2*, reinforcing the synergy in apoptosis induction. Our current study aligns with and extends these observations by confirming enhanced *Caspase-3* activation and further suppression of *Bcl-2* expression in the combination treatment group, indicating convergent mechanisms driving apoptosis. These cumulative data underline the translational potential of hesperidin as a chemosensitizer and its relevance in adjuvant cancer therapy formulations.

Although this study provides significant insights into the cytotoxic, pro-apoptotic, and immunomodulatory effects of Hes and DX in HeLa cervical cancer cells, several limitations must be acknowledged. In particular, *Bcl-2* and *Caspase-3* gene expression was not validated at the protein level, requiring caution in interpreting these transcriptional results. However, the measurement of *Caspase-3/7* enzymatic activity partially compensates for this limitation. The experiments were conducted exclusively under in vitro conditions that do not fully recapitulate the complexity of the tumor microenvironment, which may limit the translational relevance of the findings. Furthermore, this study focused on a limited cytokine panel (*IL-1β*, *IL-6*, *TNF-α*, *IFN-γ*), potentially overlooking broader immunomodulatory interactions. The fact that only the HeLa cell line was used restricts the generalizability of the results to all cervical cancer subtypes. Future work should include additional cervical cancer cell lines and normal cervical epithelial cells to evaluate cytotoxic selectivity and treatment-specific responses more comprehensively. The combination therapy was tested at multiple fixed-dose combinations, and synergy was quantitatively assessed using the Chou–Talalay method, revealing strong synergistic interactions, particularly at medium and high doses. Nonetheless, further pharmacodynamic studies using extended dose–response matrices and additional synergy models (e.g., Bliss, Loewe) would help to fully define the interaction landscape between Hes and DX. Notably, cytokine concentrations were normalized to viable cell counts and validated at the mRNA level by RT-qPCR, which strengthened the robustness of the immunomodulatory findings and reduced the likelihood that observed effects were due solely to differences in proliferation or post-transcriptional regulation.

In conclusion, this study provides compelling evidence that the combination of DX and Hes exerts a quantitatively confirmed synergistic pro-apoptotic effect on HeLa cervical cancer cells, as demonstrated by Chou–Talalay CI analysis showing strong synergy, particularly at medium and high doses. This interaction was accompanied by significant upregulation of *Caspase-3* and suppression of *Bcl-2* gene expression, indicating enhanced activation of the intrinsic apoptotic pathway. Importantly, RT-qPCR validation confirmed that the combined treatment also modulated the inflammatory profile at the transcriptional level, with marked downregulation of *IL-1β*, *IL-6*, *TNF-α*, and *IFN-γ* mRNA expression, fully consistent with ELISA results normalized to viable cell counts. This dual modulation of apoptosis and inflammation underscores the therapeutic potential of the Hes–DX combination, offering both enhanced tumor cell elimination and attenuation of chemotherapy-associated pro-inflammatory responses.

## 4. Materials and Methods

### 4.1. Cell Culture

HeLa cells (ATCC^®^ CCL-2™, American Type Culture Collection, Manassas, VA, USA), a human cervical carcinoma cell line, were cultured in Eagle’s Minimum Essential Medium (EMEM; Sigma-Aldrich, St. Louis, MO, USA) supplemented with 10% fetal bovine serum (FBS; Thermo Fisher Scientific, Waltham, MA, USA), 1% penicillin-streptomycin, and 2 mM L-glutamine (Thermo Fisher Scientific) under standard conditions (37 °C, 5% CO_2_, humidified atmosphere).

Hes (purity ≥ 98%, HPLC; Cayman Chemical, Ann Arbor, MI, USA) and DX (Sigma-Aldrich, St. Louis, MO, USA) were prepared at stock concentrations using ultrapure ethanol (Merck, Darmstadt, Germany) and sterile distilled water, respectively. Prior to treatment, working solutions of Hes were diluted in complete EMEM to final concentrations of 10, 50, 100, 250, 500, and 1000 μM. DX was applied at final concentrations of 10, 50, 100, 250, 500, and 1000 nM. All treatments were performed in triplicate, and the final ethanol concentration in all experimental and control groups did not exceed 0.1% (*v*/*v*) to avoid solvent-induced cytotoxicity.

Cells were seeded in appropriate culture vessels and allowed to adhere overnight prior to drug treatment. Control groups received culture medium containing vehicle (ethanol) without the active compound.

### 4.2. Cell Viability

The cytotoxic effects of Hes, DX, and their combination on HeLa cells were evaluated using the MTT [3-(4,5-dimethylthiazol-2-yl)-2,5-diphenyltetrazolium bromide] assay. HeLa cells were seeded at a density of 3000 cells per well in 96-well flat-bottom culture plates and allowed to adhere overnight. Cells were then treated with various concentrations of Hes (10–1000 µM), DX (10–1000 nM), or their combination. Incubation was carried out for 24, 48, and 72 h under standard conditions (37 °C, 5% CO_2_).

Following each treatment period, 10 µL of MTT solution (5 mg/mL in PBS) was added to each well, and plates were incubated for an additional 2–4 h. After the formation of purple formazan crystals, the medium was carefully removed, and 200 µL of dimethyl sulfoxide was added to each well to solubilize the formazan. The absorbance was measured at 570 nm using a microplate spectrophotometer (BioTek ELx800, BioTek Instruments, Winooski, VT, USA).

The relative cell viability (%) was calculated using the following Formula (1):

Cell viability (%) = (OD_treated_/OD_control_) × 100 (1)

Control wells (vehicle-treated) were defined as 100% viable. Each experiment was performed in triplicate, and results were expressed as mean ± standard deviation (SD). OD: Optical density.

### 4.3. Determination of IC_50_ Dose

The half-maximal inhibitory concentration (IC_50_) values of Hes, DX, and their combination were calculated based on the cell viability data obtained from the MTT assay. Dose–response curves were generated using nonlinear regression analysis with a sigmoidal dose–response (variable slope) model.

Data analysis was performed using SPSS version 20.0 (IBM Corp., Armonk, NY, USA). IC_50_ values were calculated separately for each compound and incubation period (24, 48, and 72 h) by interpolating the concentration at which 50% inhibition of cell viability was observed compared to control. All experiments were carried out in triplicate, and results were reported as mean IC_50_ ± standard deviation (SD).

### 4.4. Chou–Talalay CI Analysis

The potential synergistic, additive, or antagonistic interactions between Hes and DX were quantitatively evaluated using the Chou–Talalay method. Cell viability data obtained from the MTT assays at 24, 48, and 72 h were used to calculate the fraction affected (Fa) for each treatment dose. Fa was defined as Fa = 1 − (survival fraction), where the survival fraction corresponds to the ratio of the absorbance in the treated wells to that in the control wells.

The combination concentrations tested were low (100 µM Hes + 100 nM DX), medium (250 µM Hes + 250 nM DX), and high (500 µM Hes + 500 nM DX), selected based on the respective IC_50_ values and dose–response profiles.

The CI values were determined using CompuSyn software (version 1.0, ComboSyn Inc., Paramus, NJ, USA) based on the median-effect Equation (2):

CI = (D)_1_/(D_x_)_1_ + (D)_2_/(D_x_)_2_ (2)

where (D)_1_ and (D)_2_ represent the concentrations of drugs 1 and 2 used in combination to achieve a given Fa, and (Dx)_1_ and (Dx)_2_ are the concentrations of each drug alone required to produce the same Fa.

CI values were interpreted as follows: CI < 1 indicates synergism, CI = 1 indicates an additive effect, and CI > 1 indicates antagonism. The degree of synergism was classified according to Chou’s criteria: strong synergism (CI < 0.7), synergism (0.7 ≤ CI < 0.9), slight synergism (0.9 ≤ CI < 1.0), additive effect (CI = 1.0), and antagonism (CI > 1.0).

All CI analyses were performed in triplicate from independent experiments, and results are presented as mean ± SD.

### 4.5. Measurement of Cytokine Levels

After treatment with Hes, DX, and their combination, cell culture supernatants from HeLa cells were collected and centrifuged at 3000 rpm for 10 min at 4 °C to remove cellular debris. The clarified supernatants were aliquoted and stored at −80 °C until further analysis.

The cytokine concentrations of interleukin-1 beta (*IL-1β*; Elabscience, PKSH031840, Elabscience Biotechnology Inc., Houston, TX, USA), interleukin-6 (*IL-6*; Elabscience, E-EL-H6156), tumor necrosis factor-alpha (*TNF-α*; Elabscience, E-EL-H0109), and interferon-gamma (*IFN-γ*; Elabscience, E-UNEL-H0069) were measured using commercially available enzyme-linked immunosorbent assay (ELISA) kits according to the manufacturer’s instructions. *IL-1β*, *IL-6*, *TNF-α*, and *IFN-γ* levels were measured using commercially available ELISA kits (Elabscience Biotechnology Inc., Houston, TX, USA) in accordance with the manufacturer’s protocol. Measurements were performed using a BioTek ELx800 microplate reader at a wavelength of 450 nm. The optical density (OD) values obtained were converted to concentration units using the standard curves provided by the kits. The results were normalized according to the total protein amount in the cell supernatant and expressed as pg/mg protein or ng/mg protein. The Bradford protein determination method was used to determine the total protein amount. In addition, cytokine concentrations were further normalized to the number of viable cells (per 10^4^ cells) determined by trypan blue exclusion assay at the end of the 48-h incubation period, to account for treatment-induced differences in cell proliferation and viability. All measurements were performed in triplicate (*n* = 3) and data are reported as mean ± standard deviation (SD).

### 4.6. Caspase-3/7 Activity

*Caspase-3*/7 activity was measured quantitatively as a marker of apoptotic cell death using a commercial kit (Caspase-Glo^®^ 3/7; Promega Corporation, Madison, WI, USA). HeLa cells were seeded in 96-well plates with 3 replicates per group and incubated with Hes and/or DX at the specified concentrations for 48 h. At the end of the incubation period, reagents were added to each well according to the manufacturer’s protocol. After the plates were incubated at room temperature in the dark for 30 min, the luminescence signal was measured with a microplate reader. The values obtained were normalized to the control group and analyzed.

### 4.7. qRT-PCR Analysis

Total RNA was extracted from HeLa cells following 48 h treatments with Hes (Hes; IC_50_), DX (DX; IC_50_), and their combination using TRIzol Reagent (Invitrogen, Carlsbad, CA, USA) in accordance with the manufacturer’s instructions. RNA purity and concentration were assessed by measuring the A260/A280 absorbance ratio using a NanoDrop 2000 spectrophotometer (Thermo Fisher Scientific, USA). Only RNA samples with a ratio between 1.8 and 2.1 were used for further analysis.

Complementary DNA (cDNA) was synthesized from 1 µg of total RNA using the RevertAid First Strand cDNA Synthesis Kit (Thermo Scientific, USA) following the manufacturer’s protocol.

Quantitative real-time PCR was conducted using a SYBR Green-based detection system on the StepOnePlus Real-Time PCR System (Applied Biosystems, Foster City, CA, USA). Each reaction was performed on a final volume of 20 µL, containing SYBR Green Master Mix, gene-specific primers, and 1 µL of cDNA template. Amplification conditions were carried out according to standard protocols.

The expression levels of apoptosis-related genes *(Bcl-2* and *Caspase-3*) and inflammatory cytokines (*IL-1β*, *IL-6*, *TNF-α*, and *IFN-γ*) were evaluated. GAPDH and β-Actin were used as housekeeping genes for normalization. Each reaction was performed in triplicate, and relative gene expression was calculated using the 2^−ΔΔct^ method, with results expressed as fold change compared with untreated control cells.

Primer sequences used in this study are listed in Table 2.

### 4.8. qRT-PCR Analysis of Cytokines

Total RNA was extracted from HeLa cells treated for 48 h with Hes (IC_50_), DX (IC_50_), or their combination using TRIzol Reagent (Invitrogen, USA), as described for apoptotic gene analysis. cDNA was synthesized from 1 µg RNA using the RevertAid First Strand cDNA Synthesis Kit (Thermo Scientific, USA). RT-qPCR was performed on a StepOnePlus Real-Time PCR System (Applied Biosystems, USA) using SYBR Green Master Mix (Applied Biosystems, USA). Gene-specific primers for IL1B, IL6, TNFA, and IFNG were designed based on published sequences and validated for specificity by melt curve analysis. GAPDH served as the reference gene. Primer sequences are provided in Table 2. Reactions were run in triplicate for each of three independent biological replicates. Relative expression was calculated using the 2^−ΔΔct^ method with untreated control cells as calibrator. Statistical analysis was performed using one-way ANOVA followed by Tukey’s post hoc test, with *p* < 0.05 considered statistically significant.

### 4.9. Enrichment Analysis and Reactome Pathway Analysis

Enrichment analysis was performed to evaluate the functional involvement of the *Bcl-2* and *Caspase-3* genes in key cellular pathways. Based on Kyoto Encyclopedia of Genes and Genomes data, both genes were mapped to critical signaling cascades associated with cancer biology, particularly those regulating apoptosis and cell survival mechanisms. These pathways are highly relevant in the context of cervical cancer and provide insight into the pro-apoptotic and anti-apoptotic balance modulated in HeLa cells.

In addition, Reactome pathway analysis was conducted to further investigate the molecular events associated with these genes. Reactome is a curated, open-source database that models human biological pathways in an event-based framework. This analysis enabled the identification of specific molecular interactions and downstream processes involving *Bcl-2* and *Caspase-3*, particularly within the intrinsic apoptotic signaling pathway and caspase activation cascade. Such pathway-level insights contribute to a better understanding of the mechanisms underlying the enhanced apoptotic response observed in treated HeLa cells.

### 4.10. Gene Ontology Analysis

To assess the biological relevance of gene expression changes observed in this study, Gene Ontology (GO) analysis was performed. Following treatment of HeLa cells with DX, Hes, and their combination, total RNA was extracted and reverse-transcribed into cDNA using a high-capacity reverse transcription kit. The expression levels of apoptosis-related genes were quantified by qRT-PCR.

Differentially expressed genes were analyzed using the STRING database (https://string-db.org/, accessed on 2 July 2025) to classify them according to associated biological processes defined in the GO framework. The analysis enabled functional categorization of the target genes, particularly in relation to apoptotic regulation, immune response, and stress-induced signaling, thereby providing insight into the cellular mechanisms influenced by the treatments.

### 4.11. Statistical Analysis

Statistical analysis was performed to evaluate differences in cell viability and gene expression among treatment groups. One-way analysis of variance (ANOVA) was used to assess differences in mean cell viability (from MTT assays) and gene expression levels (from qRT-PCR). When statistically significant differences were detected, Tukey’s post hoc test was applied for multiple pairwise comparisons.

For comparisons involving two independent groups, either the independent samples *t*-test or the Mann–Whitney U test was used, depending on the normality of data distribution. All statistical analyses were conducted using SPSS version 20.0 (IBM Corp., Armonk, NY, USA). A *p*-value less than 0.05 was considered statistically significant.

## Figures and Tables

**Table 1:** Chou–Talalay CI values for Hes and DX combinations in HeLa cervical cancer cells.

Time (h)	Dose Combination (Hes + DX)	CI Value (Mean ± SD)	Interaction Type
24	100 µM + 100 nM	0.85 ± 0.04	Synergism
24	250 µM + 250 nM	0.68 ± 0.03	Strong synergism
24	500 µM + 500 nM	0.65 ± 0.02	Strong synergism
48	100 µM + 100 nM	0.78 ± 0.03	Synergism
48	250 µM + 250 nM	0.62 ± 0.02	Strong synergism
48	500 µM + 500 nM	0.60 ± 0.02	Strong synergism
72	100 µM + 100 nM	0.76 ± 0.04	Synergism
72	250 µM + 250 nM	0.61 ± 0.03	Strong synergism
72	500 µM + 500 nM	0.58 ± 0.02	Strong synergism

**Table 2:** Primer sequences used for qRT-PCR analysis.

Gene	Forward Primer (5′→3′)	Reverse Primer (5′→3′)
*Bcl-2*	ATGTGTGTGGAGAGCGTCAA	ACAGTTCCACAAAGGCATCC
*Caspase-3*	GGTATTGAGACAGACAGTGG	CATGGGATCTGTTTCTTTGC
*IL-1β*	GCAACTGTTCCTGAACTCAACT	ATCTTTTGGGGTCCGTCAACT
*IL-6*	CTGCAAGAGACTTCCATCCAG	AGTGGTATAGACAGGTCTGTTGG
*TNF-α*	CTGAACTTCGGGGTGATCGG	GGCTTGTCACTCGAATTTTGAGA
*IFN-γ*	AGACAATCAGGCCATCAGCAA	GGACCCCTGTGGGTTGTTGACC
*β-Actin*	CCTCTGAACCCTAAGGCCAAC	TGCCACAGGATTCCATACCC
*GAPDH*	CGGAGTCAACGGATTTGGTCGTAT	GCCTTCTCCATGGTGGTGAAGAC

## Data Availability

Data are contained within the article.

## Abbreviations

The following abbreviations are used in this manuscript:

Hes	Hesperidin
DX	Doxorubicin
CI	Combination Index
HPV	Human Papillomavirus
EMEM	Eagle’s Minimum Essential Medium
OD	Optical Density
IC_50_	Half-Maximal Inhibitory Concentration
IL-1β	Interleukin-1 beta
IL-6	Interleukin-6
TNF-α	Tumor Necrosis Factor-alpha
IFN-γ	Interferon-gamma
cDNA	Complementary DNA
RT-qPCR	Real-Time Quantitative Polymerase Chain Reaction
GAPDH	Glyceraldehyde-3-Phosphate Dehydrogenase
GO	Gene Ontology
MOMP	Mitochondrial Outer Membrane Permeabilization
